# Navigating Subclavian Artery Stenosis in Pregnancy: A Multidisciplinary Approach to a High-Risk Case

**DOI:** 10.7759/cureus.43933

**Published:** 2023-08-22

**Authors:** Dharmesh J Patel, Kamlesh Chaudhari, Aishwarya Gupta, Nainita Patel, Pooja P Patel

**Affiliations:** 1 Department of Obstetrics and Gynaecology, Datta Meghe Institute of Higher Education and Research, Wardha, IND; 2 Department of Dermatology, Padmashree Dr. Dnyandeo Yashwantrao Patil Medical College, Navi Mumbai, IND

**Keywords:** vascular disease, obstetric care, pregnancy complications, high-risk case, subclavian artery stenosis

## Abstract

Given the possibility of serious consequences for both the pregnant woman and the developing baby, subclavian artery stenosis (SAS) during pregnancy represents a unique but demanding scenario that requires quick and thorough treatment. In this report, we present a case of a pregnant patient with SAS who was managed effectively by employing a multidisciplinary approach, with a focus on clinical decision-making and intervention measures to ensure the best possible outcomes for both the mother and the fetus. This case report highlights the significance of prompt recognition and action to avoid the adverse consequences of SAS during pregnancy. To establish uniform standards for managing such high-risk cases and achieve better patient outcomes, more research and case studies are required.

## Introduction

Subclavian artery stenosis (SAS), a rare vascular condition, can manifest during pregnancy and poses significant risks for the mother and the developing fetus. The subclavian arteries, which are located below the clavicles, are essential for supplying blood to the head and neck as well as the upper limbs. The left subclavian artery arises from the aortic arch directly, while the brachiocephalic trunk gives rise to the right subclavian artery [1]. Chronic hypertension is the most common cause of subclavian stenosis, which might worsen during pregnancy due to increased circulatory demand and hormonal changes.

Certain significant medical problems are associated with SAS, such as ischemia consequences affecting the brain, upper limbs, and, in certain situations, the heart. While atherosclerosis is the most prevalent cause of this type of condition, radiation-induced inflammation, arteritis, fibromuscular dysplasia, compression syndromes, and neurofibromatosis are risk factors as well. Subclavian stenosis affects 3-4% of the general population [2]; however, it affects patients with peripheral arterial disease more frequently: 11-18% of this patient population at any given time [3]. When subclavian or innominate lesions are identified, 50% of patients also tend to have coronary artery disease, 27% have involvement of the lower limb arteries, and 29% have carotid obstructive disease [4,5]. This disorder thus increases the chances of symptomatic coronary artery disease and cerebrovascular events in those who are affected by it [6-8]. Often, individuals with SAS due to atherosclerotic plaques are asymptomatic; however, treatment must be provided when symptoms develop [9,10]. Upper limb ischemia symptoms tend to occur on the ipsilateral side in SAS, which is hemodynamically severe. Because of retrograde blood flow in the same-side vertebral artery, it can also result in subclavian steal syndrome (SSS), leading to vertebrobasilar insufficiency [11]. The two preferred treatments for managing symptomatic subclavian occlusive disease are angioplasty and stenting. This endovascular procedure showed a high initial success rate, with favorable outcomes lasting for more than 10 years, as per a retrospective analysis evaluating the long-term effects [12]. Early detection and management of SAS are essential for optimal maternal and fetal outcomes. In this case report, we describe a primigravida at 39 weeks and one-day gestation with known chronic hypertension who presented with SAS in the latent phase of labor. We highlight the diagnostic and management challenges of this rare but serious vascular disorder in pregnancy.

## Case presentation

**Figure 1:**
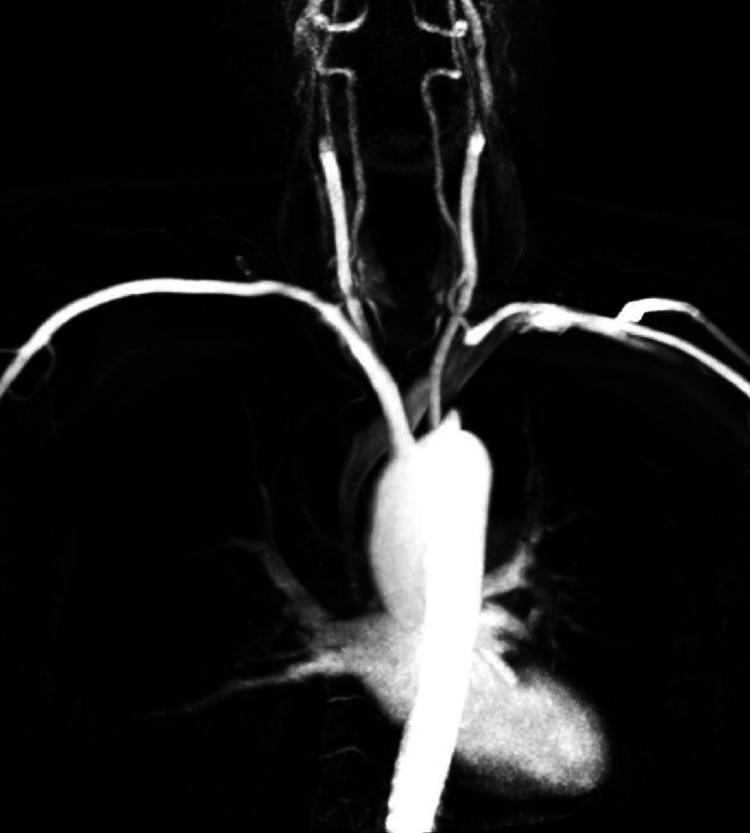
MRA showing the presence of a blockage in the proximal segment of the left subclavian artery MRA: magnetic resonance angiography

A 30-year-old primigravida at 39 weeks and one day of gestation with chronic hypertension presented to the labor and delivery unit during the latent phase of labor. The patient's medical history included well-controlled chronic hypertension with Tab. nifedipine 20 mg. Upon admission, the patient's blood pressure measured 150/90 mmHg, and the fetal heart rate was within the normal range. The obstetric ultrasound indicated a singleton fetus in cephalic presentation, estimated to weigh 3.2 kg, and no other anomalies were detected. During labor, the patient complained of numbness and tingling in her left arm. During a physical examination, it was found that her left radial pulse was low and that her right and left arms had different blood pressure readings. To exclude other possible causes, a thorough evaluation was conducted to rule out differential diagnoses such as thoracic outlet syndrome and cervical rib. A Doppler ultrasonography of the left subclavian artery demonstrated high-grade stenosis in the proximal part of the artery. After that, magnetic resonance angiography (MRA) confirmed the presence of a blockage in the left subclavian artery's proximal segment, which extended to its origin from the left vertebral artery (Figure 1).

A vascular surgeon was consulted for further evaluation. Considering her pregnancy status, the vascular surgeon recommended conservative management. However, as the patient's blood pressure remained unresponsive to oral antihypertensive medication and since she developed severe hypertension during active labor (with blood pressure reaching 170/100 mmHg), the obstetric team decided to proceed with induction of labor. Intravenous labetalol was administered, and the patient's blood pressure gradually decreased. The continuous recording of the mother's blood pressure and fetal well-being prompted the decision to proceed with a lower segment cesarean section (LSCS) delivery to minimize the risk of maternal and fetal complications. A healthy infant was born after a successful cesarean section, showing Apgar scores of 9 and 10 at one and five minutes, respectively. The patient's blood pressure was closely monitored postoperatively, and her antihypertensive medication was adjusted as needed. The vascular surgery team recommended conservative management with antiplatelet therapy and regular follow-up, including serial Doppler ultrasounds to monitor the stenosis. The patient's condition remained stable throughout her hospital stay, and she received sustained multidisciplinary care and follow-up.

## Discussion

Since clinically severe ischemia situations are less frequent in the upper extremities, lower limbs are often examined first in peripheral artery disease (PAD) assessments. Nevertheless, SAS is a unique form of PAD, potentially indicating widespread atherosclerosis and increased susceptibility to cardiovascular events. While SAS is a rare vascular disorder, it can be a significant issue during pregnancy, and chronic hypertension is a well-established risk factor for its development. Particularly when the left internal mammary artery (LIMA) is utilized as a graft during coronary artery bypass graft surgery (CABG), SAS can result in symptomatic ischemia, affecting the brain, upper limbs, and, in a few cases, the heart [13].

When the proximal subclavian artery is severely blocked, blood pressure drops beyond the occlusion. This alteration in blood flow direction causes blood to be "stolen" from the proximal branches of the artery and directed toward the distal vascular beds [14]. SSS refers to the phenomenon that causes the flow to reverse within the vertebral artery of the subclavian artery as well as the symptoms it causes. However, when SAS leads to reversed blood flow without apparent clinical symptoms, it is called the subclavian steal phenomenon [13].

Because atherosclerotic disease is more common in older people, SAS frequently manifests in this age group. Atherosclerosis affects the left subclavian artery more, with a ratio of around 3:1 to 4:1 [13,15]. This predilection for the left side is attributed to the artery's sharper origin, causing more significant turbulence and hence accelerating plaque formation. The blood pressure measurements in both arms are recorded to detect potential differences in the upper extremities in SAS patients. Additionally, carotid, cervical, or supraclavicular bruits are examined. Finger ulcers, necrosis, splinter hemorrhages, or gangrenous skin changes are less common physical findings. The carotid or subclavian arteries can produce bruits that can be auscultated to detect hidden underlying problems.

Duplex ultrasonography with color flow imaging is the best noninvasive method for evaluating subclavian artery disease. Waveforms that are dampened or monophasic, turbulent color flow imaging, and greater velocity in the stenotic area are common signs of obstruction. The ipsilateral vertebral artery's flow is reversed, which is a sign of SSS. CT angiography offers exceptional anatomical resolution, providing valuable insights into the length and location of the lesion [16]. However, it may not provide the best insight into the level of calcification.

Additionally, digital subtraction angiography and fluoroscopy cannot precisely quantify the degree of calcification. MRA may sometimes lead to misinterpretation due to reduced flow, potentially leading to overestimating the disease severity. The definitive test for assessing subclavian artery disease is invasive angiography. This procedure enables detailed anatomical mapping without subtracted images and provides additional information on stenosis using digital subtraction techniques. However, it is essential to highlight that there is a slight risk of stroke (less than 1%) associated with manipulating the aortic arch and brachiocephalic arteries during the angiography procedure.

The treatment approach for SAS is determined based on the severity of the stenosis and the specific symptoms observed in the patient. Conservative management with medications such as antiplatelets and anticoagulants may be sufficient in mild cases, but more severe cases may require surgical intervention. Options for surgical intervention include angioplasty, stent placement, or even surgical bypass. Intervention selection will be contingent on factors such as the location and extent of the stenosis and the presence of any concurrent medical conditions. Due to the potential hazards to the mother and the fetus, managing SAS presents substantial complications during pregnancy. To reduce injury to both, weighing the risks and advantages of various therapeutic approaches is crucial. Doppler ultrasound and MRA were used to diagnose left SAS in our patient, a 39 weeks one-day pregnant primigravida with documented chronic hypertension in the latent phase of labor. Anticoagulants and antiplatelets were used to manage the patient conservatively, and she underwent an emergency LSCS without any adverse effects on her or the newborn. Early detection and efficient management are essential to avoid severe consequences and improve outcomes for both the mother and the fetus. To reduce the risks to both the mother and the fetus, healthcare practitioners must be watchful about this condition, acquaint themselves with the available treatment options, and apply careful judgment in its management.

## Conclusions

This case study underscores the importance of considering SAS in pregnant individuals with chronic hypertension, as it can result in significant maternal and fetal complications. Timely identification and appropriate handling, involving a meticulous assessment of the pros and cons of various treatment choices, play a pivotal role in achieving favorable outcomes for the mother and fetus. Furthermore, this report highlights the need for healthcare practitioners to be aware of this infrequent yet potentially critical vascular condition and be familiar with the available treatment options. These options encompass conservative approaches utilizing medications or more proactive interventions like angioplasty, stent insertion, or surgical bypass. In summary, this report emphasizes the significance of early recognition and effective management of SAS in pregnant women with chronic hypertension. Such vigilance mitigates serious complications and leads to excellent outcomes in terms of maternal and neonatal well-being.
